# The impact of maternal adverse childhood experiences on children's quality of life: the moderating role of self-esteem and the mediating role of maternal quality of life

**DOI:** 10.3389/fgwh.2025.1630244

**Published:** 2025-10-20

**Authors:** Eunjeong Cho, Yeon Jeong Heo, Eunha Ryoo, Hye Jin Kim

**Affiliations:** 1 Department of Nursing, Nursing Research Institute, Hallym Polytechnic University, Chuncheon, Republic of Korea; 2 Department of Nursing, Dongnam Health University, Suwon, Republic of Korea; 3 Department of Nursing, Changshin University, Changwon, Republic of Korea

**Keywords:** maternal, adverse, childhood, experiences, quality of life, self-esteem, intergenerational transmission

## Abstract

**Introduction:**

This study examined the impact of maternal adverse childhood experiences (ACEs) on their children's overall quality of life (QoL), focusing on the mediating role of maternal QoL and the moderating role of maternal self-esteem. Understanding these intergenerational pathways can provide valuable insights for designing interventions that promote family well-being.

**Methods:**

A secondary data analysis was conducted using data from the 2018 National Child Life Experience Survey in South Korea. Participants included 930 mothers who had experienced at least one type of childhood adversity. A descriptive and correlational research design was employed, and statistical analyses were performed using the PROCESS macro to test mediation and moderation effects.

**Results:**

Higher levels of maternal ACEs were significantly associated with lower QoL in both mothers and their children. Maternal QoL partially mediated this relationship, indicating that adverse childhood experiences affect children's well-being indirectly through maternal health. Moreover, maternal self-esteem moderated the negative effects of maternal ACEs on children's QoL, serving as a psychological protective factor.

**Discussion:**

These findings highlight the critical importance of maternal psychological health in mitigating the intergenerational transmission of adversity. Strengthening maternal self-esteem and emotional well-being could buffer the negative impact of early adversity on families. Public health and nursing strategies that integrate mental health promotion and family-based interventions are essential to improve long-term outcomes for children in families affected by maternal ACEs.

## Highlights

- Maternal adverse childhood experiences (ACEs) negatively affect both maternal and child quality of life (QoL).
- Maternal QoL mediates the relationship between maternal ACEs and children's QoL, reflecting intergenerational transmission.
- Maternal self-esteem moderates the negative impact of ACEs on children's QoL, serving as a protective factor.
- Enhancing maternal self-esteem and psychological well-being may reduce the adverse effects of ACEs.
- The findings underscore the need for nursing and public health interventions to support maternal mental health and family well-being.

## Background

According to Bronfenbrenner's (1) ecological systems theory, child development occurs through interactions among multiple environmental systems surrounding the individual (e.g., microsystem, mesosystem, exosystem, and macrosystem). Parents constitute a core element at the center of the child's microsystem, and parents' adverse childhood experiences (ACEs) can affect their children's development and quality of life (QoL) across generations (1–3).

Maternal ACEs refer to severe stressors experienced before age 18 such as abuse, neglect, and family discord (4) that can leave psychosocial sequelae into adulthood and exert long-term effects on mothers' mental health and parenting behaviors (5). Recent studies report that higher parental ACE scores are significantly associated (6) with increased risks of children's emotional/behavioral problems and overall health problems (3). In a large-scale study, Chen et al. (5) found that 85.8% of Chinese mothers reported at least one ACE, and even a single ACE in mothers was linked to significantly lower child health-related QoL. Such intergenerational transmission of adversity has been observed across diverse cultures, and elucidating its mechanisms is of scholarly and practical importance (7–9).

Mothers' psychosocial status is a key pathway mediating the impact of ACEs on children. When mothers experience depression, anxiety, or emotion-regulation difficulties related to past ACEs, their current QoL and mental health decline, leading to increased parenting stress and reduced parenting responsiveness, which ultimately undermines children's development and well-being (5, 10–12). In a Canadian longitudinal study, Letourneau et al. (12) showed that prenatal/postpartum depression and anxiety mediated the effects of maternal ACEs on children's problem behaviors. An Irish study by Swords et al. (13) likewise identified maternal mental health deterioration and poorer mother–child relationship quality as important mediators explaining the intergenerational effects of ACEs.

Maternal QoL a composite indicator encompassing physical and psychological health, social support, and relationship satisfaction is closely tied to parenting capacity. The World Health Organization (14) defines QoL as a multidimensional concept reflecting individuals' perceptions of their position in life in relation to their goals, expectations, standards, and concerns. Low QoL can reduce emotional availability and consistency in parenting and diminish responsiveness, adversely affecting children's emotional stability and social development (15, 16). Empirically, Chen et al. (5) suggested that maternal ACEs may negatively influence children's QoL through decrements in maternal QoL.

Maternal self-esteem, meanwhile, is highlighted as a protective factor that can buffer such adverse effects. Self- esteem refers to the evaluation of one's own worth, and women with ACE histories tend to exhibit lower self- esteem (17–19). Mothers with higher self-esteem generally report greater parenting efficacy and demonstrate warm, consistent parenting behaviors (20, 21); higher parental self-esteem is also associated with increased self-esteem and happiness in children (22). This evidence suggests that maternal self-esteem may moderate the association between ACEs and children's QoL, such that higher self-esteem mitigates the negative impact of past adversity.

Although theoretical and empirical work has accumulated on the links between parental ACEs and child development, integrated research is scarce on how psychosocial resources such as QoL and self-esteem jointly mediate and moderate the effects of ACEs. From an ecological perspective, maternal QoL and self-esteem are core elements within the family microsystem that interact with macrosystem factors (e.g., socioeconomic conditions and cultural values) to influence child development. For mothers, ACEs originate in the childhood home (microsystem) yet persist across the chronosystem into adulthood; for children, the mother's psychosocial status functions as an exosystem factor that indirectly affects development (1). Accordingly, grounded in an ecological model, this study simultaneously examines the mediating role of maternal QoL and the moderating role of maternal self-esteem in the association between maternal ACEs and children's QoL, with the goal of specifying intergenerational pathways of adversity and informing prevention strategies.

### Theoretical framework

This study is theoretically grounded in Bronfenbrenner's (1) ecological systems theory, which posits that human development occurs through dynamic interactions between individuals and the multiple environmental systems surrounding them. These systems include the microsystem, mesosystem, exosystem, macrosystem, and chronosystem, each influencing and interacting with one another throughout the developmental process.

From this perspective, a mother's ACEs can be considered a distal risk factor originating in the chronosystem and early microsystem. ACE can leave enduring psychological and social consequences into adulthood, particularly affecting a mother's QoL and psychosocial resources such as self-esteem. These resources, in turn, have direct and indirect impacts on the development and well-being of her children.

In the conceptual model of this study, the mother's QoL is conceptualized as a mediating variable reflecting current psychosocial functioning. It is hypothesized that the effects of ACE on maternal mental health, physical health, social relationships, and life satisfaction are transmitted to children through QoL. Conversely, maternal self-esteem is posited as a protective factor buffering the intergenerational transmission of adversity. Mothers with high self-esteem are more likely to maintain parenting efficacy, remain consistent, and engage in positive parenting behaviors even under stressful circumstances.

This study also incorporates socio-ecological covariates, as proposed in Bronfenbrenner's model. For example, variables such as education level, household income, and perceived health status classified within the exosystem or macrosystem interact with mothers' internal resources (QoL, self-esteem) and influence child development. By statistically controlling for these covariates, the study aims to isolate the unique effects of maternal psychosocial characteristics on children's QoL.

**Figure 1:**
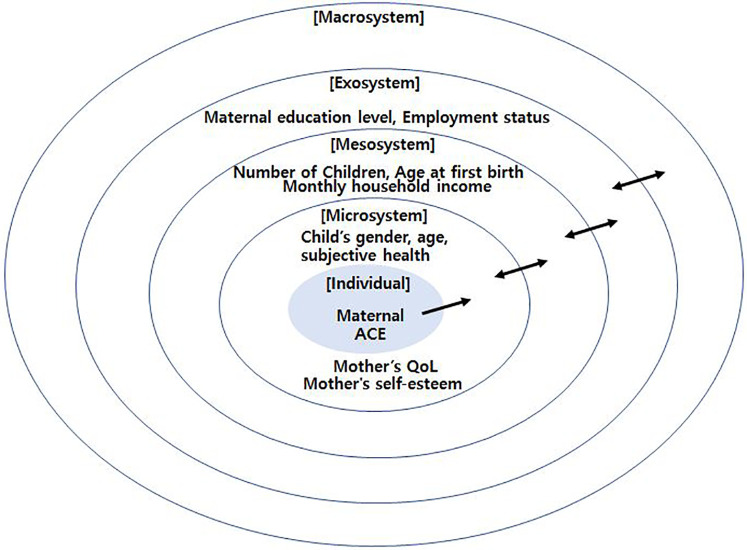
Conceptual framework of the study based on Bronfenbrenner's ecological systems theory.

In conclusion, the theoretical framework of this study presents an integrative model that explains how ACE, QoL, self-esteem, and socio-environmental factors interact within the multilayered structure of the ecological system to influence children's QoL. This framework not only elucidates the pathways of intergenerational transmission of adversity but also provides scientific evidence for designing multi-level intervention strategies. Figure 1 illustrates the conceptual framework of this study, adapted from Bronfenbrenner's ecological systems theory. It visually depicts the multiple nested systems individual, microsystem, mesosystem, exosystem, and macrosystem showing how maternal ACE, QoL, and self-esteem are embedded within and influenced by broader socio-environmental contexts. The diagram highlights the directional relationships between these variables and situates them within the ecological layers, clarifying the hypothesized pathways through which maternal characteristics and contextual factors impact children's QoL.

### Current status and aim

Although maternal ACEs have been widely recognized as risk factors for adult psychological distress and maladaptive parenting, their intergenerational impact on children's QoL remains underexplored. Specifically, few studies have examined the mechanisms through which maternal ACEs influence children's QoL, particularly in relation to maternal psychosocial resources such as quality of life and self-esteem.

Previous studies have often treated parental ACEs as static background variables, overlooking the dynamic roles of maternal well-being and internal psychological factors. Moreover, inconsistent findings remain regarding the extent to which maternal self-esteem serves as a protective factor, and limited research has empirically tested both mediation and moderation models in a single analytic framework.

Therefore, this study aims to investigate how maternal QoL mediates the relationship between maternal ACEs and children's QoL, and whether maternal self-esteem moderates this relationship. By clarifying these pathways, the study seeks to enhance the understanding of intergenerational transmission of adversity and inform targeted intervention strategies that support maternal psychological health to improve child outcomes.

## Methodology and materials

### Study design

The study was a descriptive, correlational research conducted to verify the effects of mothers' ACEs and QoL on the QoL of their children and validate the moderating effect of mothers' self-esteem and the mediating effect of QoL.

### Setting and participants

This secondary data analysis utilized raw data from the 2018 Childhood Life Experience Survey conducted by the Korea Institute for Health and Social Affairs. A two-stage stratified sampling method was applied to select survey districts and households to ensure the representativeness of the sample.

In the sample extraction process, enumeration districts were selected based on the results of the Population and Housing Census, and samples were allocated based on adults aged 19–59. Subsequently, the systematic sampling method was used to select households with children aged 9 to under 18 in each enumeration district for the survey, thereby constructing nationally representative data (23).

From the total of 1,515 respondents, the present study specifically targeted married mothers with an ACE score of one or more. The inclusion of only married mothers was a deliberate methodological choice aimed at ensuring comparability in family structure and controlling for potential confounding effects of marital status on maternal psychological resources, parenting behaviors, and child outcomes. Restricting the sample to mothers living with their spouse and children allowed for a clearer examination of the intergenerational transmission of adversity within relatively stable family environments. Respondents with missing values in any of the variables used in the analyses were excluded. The final analytic sample comprised 930 participants.

Children's QoL was assessed through self-report questionnaires completed directly by children aged 9–17 years. In households with multiple children, each eligible child within this age range was invited to respond to the child survey, rather than a pre-designated “target child”.

## Measurements

### Demographic characteristics

The general characteristics of the study participants were classified as maternal age, education level, dual- income status, average monthly income, subjective health status, number of children, age at first childbirth, child gender, child age, and child's subjective health status. Maternal age was categorized as under 40, in the 40s, and over 50. Educational level was categorized as high school or below and college or above. Dual-income status was categorized as single-income and dual-income. Average monthly income and maternal subjective health status were categorized as poor, average, good, and very good. The number of children was categorized as one, two, three, and four. Children's gender was categorized as male and female. Children's ages were categorized as under 13 years old or 13 years or older. Children's subjective health status was categorized as poor, average, good, and very good.

### Adverse childhood experiences

The Adverse Childhood Experiences International Questionnaire (ACE-IQ) is a scale used to assess the impact of ACEs on adult life (24). It employs a retrospective self-reporting approach for adults, reflecting on experiences before the age of 18. The questionnaire includes 13 items covering emotional abuse, physical abuse, sexual abuse, emotional neglect, physical neglect, family violence, and other family issues. According to guidance for analyzing ACE-IQ, any experience reported at least once is recorded as “present” (1), and no experience as “absent” (0). The scores of each item were summed, with a possible range from 1–13 points. A higher score indicates a greater number of adverse childhood experiences.

### Maternal self-esteem

Self-esteem was measured using the 10-item Rosenberg Self-Esteem Scale. The self-esteem scale includes statements such as “I feel that I am at least as worthy as other people”, “I feel that I have a number of good qualities”, and “I tend to feel that I am a failure”. The respondents indicated how much they agreed with each statement, and the responses were categorized into four levels (1 = strongly disagree, 2 = disagree, 3 = agree, 4 = strongly agree). Negatively worded items were reverse-coded to calculate the total score for each item. A higher score indicates higher self-esteem. Cronbach's alpha for self-esteem measured in this study was.69.

### Maternal QoL

The overall QoL was measured using seven items. It assesses self-reported satisfaction with life and personal circumstances, considering aspects such as standard of living, health, achievements in life, personal relationships, feelings of safety, sense of community belonging, and security about the future. The responses for each item are categorized on a scale from 0 (not satisfied at all) to 10 (very satisfied). A higher score indicates a higher level of QoL. Cronbach's alpha for QoL measured in this study was.89.

### Children's QoL

QoL was measured using six items. It assesses self-reported overall satisfaction with life through statements such as “I enjoy my life”, “My life is going well”, “I am living a good life”, “Many good things happen in my life”, “I like my life”, and “I feel happiness about my life”. The responses for each item are categorized on a scale from 0 (strongly disagree) to 10 (strongly agree). A higher score indicates a higher level of QoL. Cronbach's alpha for QoL measured in this study was .95.

## Data collection and analysis

### Data analysis

The negative impact of maternal ACEs on both maternal and child QoL was analyzed, as well as the transmission processes involved. Additionally, the moderating effect of maternal self-esteem in this relationship was examined using SPSS 26 and the PROCESS macro program. Initially, the demographic characteristics of the subjects were determined through frequency and descriptive statistical analysis. Pearson correlation analysis was conducted to assess the characteristics of the main variables and their interrelationships. Analyses using Models 4 and 5 of the PROCESS macro were performed to validate the research model. The significance of the mediating effects and conditional direct effects was confirmed using bootstrapping tests. The significance of the mediating effects and conditional direct effects was confirmed using bootstrapping tests. PROCESS model 4 (mediation) and model 5 (moderation) were selected to examine the hypothesized mechanisms separately. While models 8 and 59 allow simultaneous estimation of mediated moderation or moderated mediation, they assume a reciprocal and dynamic interplay between maternal self-esteem and maternal QoL. Conceptually, self-esteem and QoL are indeed interrelated; however, our theoretical framework, grounded in Bronfenbrenner's ecological model, positioned maternal QoL as an intrapersonal resource shaped by ACEs, and self-esteem as a personal protective factor moderating this pathway. Therefore, we intentionally tested the two processes independently to preserve model parsimony, avoid overfitting given sample size limitations, and to align with the conceptual model of unidirectional influence hypothesized in this study.

## Results

### General characteristics of the study population

A frequency analysis was conducted to examine the general characteristics of the study subjects (Table 1). The maternal age distribution showed that 205 participants (22.0%) were under 40 years old, 636 (68.4%) were in their 40s, and 89 (9.6%) were over 50, with an average age of 43.37 years. Four hundred and forty-nine (48.3%) had high school educations or below and 481 (51.7%) had college or above. Regarding employment status, 371 participants (39.9%) were in single-income families, while 559 participants (60.1%) were in dual-income families. The average monthly income was 4,801,800 KRW (approximately 3,693.69 USD as of March 4, 2025). Maternal subjective health levels were reported as poor by 10 participants (1.1%), average by 187 participants (20.1%), good by 634 participants (68.2%), and very good by 99 participants (10.6%). Of the participants, 269 (28.9%) had one child, 479 (51.5%) had two children, 161 (17.3%) had three children, and 21 (2.3%) had four or more. The average maternal age at the birth of the first child was 28.61 years.

**Table 1:** Participant characteristics and descriptive statistics (*N* = 930).

Characteristics	Categories	*N*	Weighted %	Mean ± SD
Maternal age	<40	205	22.0	43.37 ± 4.79
	40–49	636	68.4	
	≥50	89	9.6	
Maternal education level	High school or higher	449	48.3	
	College or higher	481	51.7	
Employment status	Single income	371	39.9	
	Dual income	559	60.1	
Monthly household income (unit 10,000 KRW)				480.18 ± 145.84
Maternal subjective health	Poor	10	1.1	
	Fair	187	20.1	
	Good	634	68.2	
	Very Good	99	10.6	
Number of Children	1	269	28.9	
	2	479	51.5	
	3	161	17.3	
	4	21	2.3	
Age at first birth				28.61 ± 3.85
Child's gender	Male	482	51.8	
	Female	448	48.2	
Child's age	Under 13 years old	396	42.6	13.16 ± 2.72
	13 years old and above	534	57.4	
Child's subjective health	Poor	2	0.2	
	Fair	63	6.8	
	Good	496	53.3	
	Very good	369	39.7	
ACE				3.62 ± 1.89
Maternal QoL				7.18 ± 0.93
Child QoL				7.19 ± 1.21
Maternal self-esteem				2.96 ± 0.35

SD*,* standard deviation; KRW, Korean won; ACE, adverse childhood experiences; QOL, quality of life.

Examining the characteristics of the children, 482 (51.8%) were male and 448 (48.2%) were female. Of them, 396 (42.6%) were aged 12 or below, and 534 (57.4%) were aged 13 or above, with an average age of 13.16 years. Children's subjective health status was reported as poor by two children (.2%), average by 63 children (6.8%), good by 496 children (53.3%), and very good by 369 children (39.7%). The levels of adverse childhood experiences, self-esteem, quality of life, and children's quality of life among the study participants were as follows (Table 2). The average for adverse childhood experiences was 3.62 ± 1.89, for self-esteem 2.96 ± 0.35, for quality of life 7.18 ± 0.93, and for children's quality of life 7.19 ± 1.21.

**Table 2:** Pearson's correlation matrix among ACE, QoL, self-esteem, and child QoL (*N* = 930).

Variables	1	2	3	4
	*r* (*p*)	*r* (*p*)	*r* (*p*)	*r* (*p*)
1. ACE	1			
2. Maternal QOL	−.184***	1		
3. Child QOL	−.204***	.470***	1	
4. Maternal self-esteem	−.102**	.316***	.318***	1

ACE, adverse childhood experiences; QOL, quality of life.

* *p* < .05.

** *p* < .01.

*** *p* < .001.

### Correlation between participants' ACEs, self-esteem, QoL, and their children's QoL

ACEs were found to have a significant negative correlation with maternal QoL (*r* = −.184, *p* < .001), children's QoL (*r* = −.204, *p* < .001), and maternal self-esteem (*r* = −.102, *p* < .01). Conversely, maternal QoL showed a significant positive correlation with children's QoL (*r* = .470, *p* < .001) and self-esteem (*r* = .316, *p* < .001). Children's QoL also demonstrated a significant positive correlation with maternal self-esteem (*r* = .318, *p* < .001) (Table 2). Descriptive statistical analysis of the main variables confirmed that the skewness and kurtosis did not exceed the absolute values of 3 and 10, respectively, indicating that the data met the criteria for normal distribution (25).

### The impact of ACEs on children's QoL mediated by the quality of life of the participants

The pathways through which maternal ACEs impacted their QoL, and subsequently their children's QoL, were analyzed using Model 4 of the PROCESS macro. The control variables included demographic characteristics such as maternal age, education, dual-income status, income, subjective health, number of children, age at first childbirth, child's gender, age, and subjective health (Table 3). The first model assessed the impact of the independent variable (ACE) on the mediator (maternal QoL) and showed significant results. The *F*-value indicated that this model explained 16.8% of the variance. The analysis revealed that, even after controlling for demographic characteristics, ACE had a significant negative impact on maternal QoL (*β* = −.177, *p* < .001), indicating that higher levels of ACE were associated with lower QoL.

**Table 3:** Regression results for the mediation model (PROCESS model 4) (*N* = 930).

Model	Dependent Variable	Independent Variable	B	SE	*β*	T	p	F(R^2^)
1	Maternal QOL	ACE	−0.086	0.015	−0.177	−5.817***	0.000	16.803*** (.168)
		Maternal age	0.012	0.010	0.064	1.235	0.217	
		Education level	0.192	0.059	0.104	3.257**	0.001	
		Employment status	0.103	0.060	0.054	1.719	0.086	
		Income	0.001	0.000	0.126	3.893***	0.000	
		Maternal subjective health	0.305	0.053	0.191	5.778***	0.000	
		Number of children	0.073	0.040	0.059	1.822	0.069	
		Age at first birth	−0.014	0.010	−0.057	−1.365	0.173	
		Child's gender	−0.060	0.056	−0.033	−1.072	0.284	
		Child's age	−0.013	0.015	−0.039	−0.899	0.369	
		Child's subjective health	0.222	0.050	0.145	4.417***	0.000	
2	Child QOL	ACE	−0.076	0.018	−0.119	−4.234***	0.000	35.590*** (.318)
		Maternal QOL	0.504	0.039	0.386	12.909***	0.000	
		Maternal age	0.004	0.012	0.014	0.298	0.766	
		Education level	0.248	0.070	0.103	3.537***	0.000	
		Employment status	0.031	0.071	0.013	0.434	0.665	
		Income	0.001	0.000	0.057	1.924	0.055	
		Maternal subjective health	−0.150	0.063	−0.072	−2.367*	0.018	
		Number of children	−0.021	0.048	−0.013	−0.449	0.654	
		Age at first birth	0.006	0.012	0.018	0.462	0.644	
		Child's gender	0.129	0.066	0.054	1.946	0.052	
		Child's age	−0.047	0.018	−0.106	−2.679**	0.008	
		Child's subjective health	0.463	0.060	0.232	7.718***	0.000	
3	Child QOL	ACE	−0.119	0.019	−0.187	−6.249***	0.000	20.058*** (.194)
		Maternal QOL	0.010	0.013	0.038	0.759	0.448	
		Maternal education level	0.344	0.076	0.142	4.552***	0.000	
		Employment status	0.083	0.077	0.034	1.074	0.283	
		Monthly household income	0.001	0.000	0.106	3.312**	0.001	
		Mother's subjective health	0.003	0.068	0.002	0.047	0.962	
		Number of children	0.016	0.052	0.010	0.300	0.764	
		Age at first birth	−0.001	0.013	−0.005	−0.110	0.913	
		Child's gender	0.099	0.072	0.041	1.372	0.171	
		Child's age	−0.054	0.019	−0.121	−2.819**	0.005	
		Child's subjective health	0.574	0.064	0.288	8.911***	0.000	

QoL, quality of life; ACE, adverse childhood experiences; B, unstandardized coefficient; SE, standard error; β, standardized coefficient.

* *p*  < .05.

** *p* < .01.

*** *p* < .001.

The second model analyzed the effects of both the independent variable and the mediator on the dependent variable (children's QoL), again showing a significant F-value and explaining 31.8% of the variance. The results again found that ACEs had a significant negative impact (*β* = −.119, *p* < .001), while maternal QoL had a significant positive impact (*β* = .386, *p* < .001). These findings suggest that higher maternal ACE levels correlate with lower children's QoL, whereas higher maternal QoL corresponds with higher children's QoL.

The third model explored the direct relationship between the independent variable and the dependent variable, showing significant results, with an *F*-value and 19.4% of the variance explained. ACEs were found to have a direct negative impact on children's QoL (*β* = −.187, *p* < .001). The significance of both the direct and mediated effects indicates partial mediation. The significance of the mediating effect was further confirmed by the bootstrapping test, which showed that the confidence interval did not include zero, verifying that maternal ACEs indirectly affect children's QoL through maternal QoL (Table 4).

**Table 4:** Bootstrapping results for mediation effect.

Pathway	B	S.E.	95% CI	
			95% CI (Lower)	95% CI (Upper)
ACE → Maternal QOL → Child QOL	−0.0435	0.0087	−0.0612	−0.0268

S.E., standard error; 95% CI, 95% confidence interval; LLCI, lower limit of the confidence interval; ULCI, upper limit of the confidence interval.

### The moderating effect of maternal self-esteem on the relationship between participants' ACEs and their children's QoL

**Figure 2:**
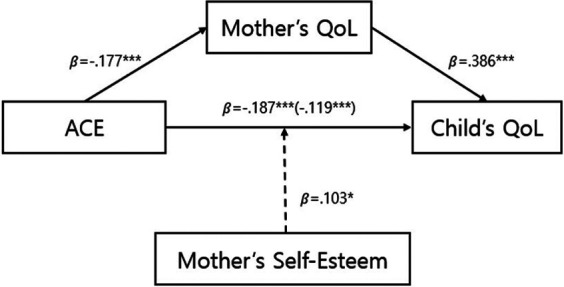
Proposed conceptual model of relationships among maternal ACEs, maternal QoL, maternal self-esteem, and children's QoL.

**Figure 3:**
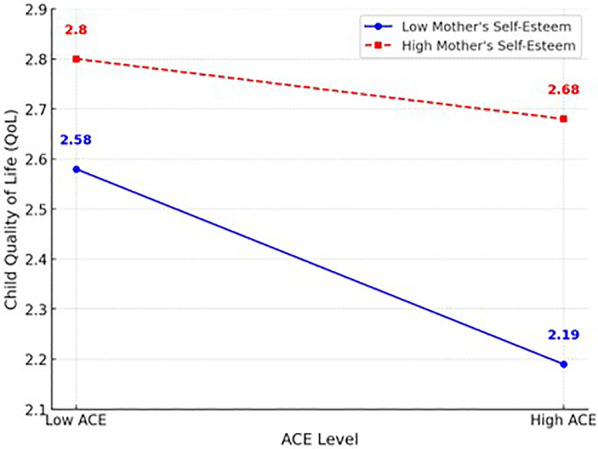
Moderation model of the effect of maternal self-esteem on the relationship between maternal ACEs and child QoL.

The moderating effect of maternal self-esteem on the pathway through which maternal ACEs impact children's QoL was analyzed using Model 5 of the PROCESS macro, as shown in Table 5. This model also controlled for the demographic characteristics of both the mothers and the children and showed significant results, with an F- value and a high explanatory power of 33.8%. The analysis revealed that ACEs had significant negative impacts on children's QoL (B = −.068, *p* < .001), while both maternal QoL (B = .458, *p* < .001) and maternal self-esteem (B = .506, *p* < .001) had significant positive effects on children's QoL. Notably, the interaction term ACE maternal self-esteem also showed a significant positive impact (B = .103, *p* < .05), indicating that high maternal self-esteem can mitigate the negative effects of ACEs on children's QoL. In the negative relationship shown as decreases in children's QoL by maternal ACEs, maternal self-esteem acted as a protective factor. Figure 2 shows the results of a simple slope test. The solid line in Figure 3 illustrates low maternal self-esteem, where higher levels of ACEs are significantly associated with lower child QoL. In contrast, the dotted line represents high maternal self-esteem, where the negative association between ACEs and child QoL is attenuated and not statistically significant.

**Table 5:** Moderation effect of maternal self-esteem on the ACE–child QoL relationship (PROCESS model 5) (*N* = 930).

DV	IV	B	SE	*t*	*p*	F(R^2^)
Child QOL	ACE	−0.068	0.018	−3.866***	0.000	33.419*** (.338)
	Maternal QOL	0.458	0.040	11.578***	0.000	
	Maternal self-esteem	0.506	0.101	4.985***	0.000	
	ACE x Maternal self-esteem	0.103	0.047	2.195*	0.028	
	Maternal Age	0.003	0.012	0.232	0.816	
	Maternal education level	0.206	0.069	2.967**	0.003	
	Employment status	0.063	0.070	0.893	0.372	
	Monthly household income	0.000	0.000	1.547	0.122	
	Mother's subjective health	−0.155	0.063	−2.480*	0.013	
	Number of children	−0.033	0.047	−0.703	0.482	
	Age at first birth	0.004	0.012	0.315	0.753	
	Child's gender	0.135	0.065	2.068*	0.039	
	Child's age	−0.048	0.017	−2.741**	0.006	
	Child's subjective health	0.428	0.060	7.195***	0.000	

* *p* < .05.

** *p* < .01.

*** *p* < .001.

Among the covariates, maternal education was significantly associated with higher child QoL. This finding suggests that contextual factors such as maternal education level may play an important role in shaping child outcomes, beyond the effects of maternal ACEs, QoL, and self-esteem.

## Discussion

This study examined the effects of maternal ACE on children's QoL and tested the mediating effect of maternal QoL and the moderating effect of maternal self-esteem in this relationship. The results indicated that higher maternal ACE scores were significantly associated with lower maternal QoL and lower children's QoL. Maternal QoL partially mediated the relationship between ACE and children's QoL, and maternal self-esteem functioned as a protective factor that mitigated the negative effects of ACE. These findings suggest that QoL is not merely a psychological state but a concept encompassing mothers' functional capacities, emotional health, and the quality of relationships. This underscores the importance of maternal QoL for effective parenting and child well-being, consistent with the World Health Organization's (WHO) definition of QoL as an integrated concept of physical, mental, and social well-being (26, 27).

There is a mutually reinforcing, bidirectional relationship between QoL and self-esteem. Mothers with higher self-esteem are more likely to have a sense of control and purpose in life, leading to enhanced QoL; in turn, higher QoL fosters greater self-evaluation and resilience (22, 28). Ultimately, maternal psychological health plays a critical role in child development, and improving maternal self-esteem can serve as a strategy to prevent the intergenerational transmission of adversity (29).

However, since our study was cross-sectional, we could not perform dynamic tests of simultaneous associations between maternal QoL and self-esteem. Future longitudinal research should examine their reciprocal influences over time to confirm this bidirectional relationship.

The associations revealed in this study are generally consistent with prior research. Previous studies have emphasized that parents' psychological stability and positive life experiences are essential for their children's well-being (30–33). In families affected by ACE, the need to strengthen emotional support and enhance resilience has been consistently reported (34). This highlights the importance of interventions that foster emotional support networks and personal resilience among parents with adversity histories for promoting healthy child development.

Specifically, studies have shown that emotional support programs can enhance mothers' resilience and prevent behavioral and emotional problems in children (29). Other research has found that low maternal life satisfaction mediates the relationship between ACE and adverse child outcomes. In other words, mothers with high ACE exposure tend to have lower life satisfaction, which in turn is associated with poorer child emotional regulation and prosocial behavior (29, 35). While factors such as resilience, emotional support, and life satisfaction have been discussed individually, they are ultimately closely interconnected within the broader framework of QoL. QoL integrates these components as a comprehensive indicator of well-being, reflecting how environmental adversity or support influences functional outcomes. This study empirically confirmed that maternal QoL functions as a mediator absorbing and reflecting the psychosocial impacts of ACE. By identifying these patterns in a nationally representative Korean sample, this research complements international studies on the negative effects of parental ACE on children within the domestic context (8, 36, 37).

In line with Bronfenbrenner's ecological systems theory (1), these results illustrate that a distal maternal risk factor (ACEs) can adversely affect a mother's internal resources (QoL, self-esteem), which in turn influences her child's QoL within the family microsystem. This supports the notion that parents' internal psychological factors are key mediating and moderating mechanisms in the intergenerational transmission of risk. For example, mothers with higher self-esteem may perceive parenting challenges as more manageable and maintain positive parenting behaviors, thereby buffering their children from the adverse impacts of maternal ACEs (20). At the same time, broader contextual factors were also significant: higher maternal education, better self-rated health, and greater household income were each associated with higher QoL for both mothers and children. This finding underscores that beyond individual traits, socio-economic conditions in the exosystem and macrosystem also shape child well-being. Therefore, improving maternal psychosocial well-being and addressing contextual disadvantages may help protect the healthy development of the next generation. In addition, a randomized controlled trial in Korea demonstrated that a mindful mothering intervention program effectively improved maternal self-efficacy and reduced the negative psychosocial impacts of ACE (38). This evidence suggests that targeted, strengths-based interventions can mitigate intergenerational risks and directly enhance maternal psychological resources, thereby reinforcing the practical implications of our findings.

### Implications

These findings have several practical implications for intervention and policy. At the individual and family level, efforts should focus on enhancing maternal self-esteem and mental well-being. Evidence-based programs such as cognitive-behavioral therapy and parenting interventions (e.g., the Triple P program) have been shown to improve mothers' psychological health, parenting skills, and mother–child relationships (20, 39–41). At the community level, expanding access to maternal mental health services and parent support programs (such as peer support groups or home visitation for at-risk families) is critical (36). Healthcare and educational professionals should also be trained to recognize signs of parental trauma and connect families with appropriate resources for early intervention.

At the policy level, broader structural supports and preventive measures are needed. Raising public awareness about the long-term impacts of ACEs and integrating ACE screening into routine prenatal and pediatric care could facilitate early identification of high-risk families (CDC, 2025); (8, 42, 43). Additionally, implementing socio-economic support policies (e.g., financial assistance, childcare subsidies, and affordable mental health services) alongside family-friendly workplace arrangements (e.g., flexible hours, parental leave) would help alleviate stress on vulnerable parents. Finally, strengthening social services and legal protections to prevent child abuse and domestic violence remains imperative to safeguard at-risk children. Taken together, a comprehensive multi-level approach addressing both individual psychological resources and broader socio-economic factors is needed to effectively break the intergenerational cycle of adversity and improve children's QoL (8, 36). Relevant institutions should provide tailored support programs for high-risk mothers.

### Limitations

This cross-sectional study limits the ability to determine causal relationships between maternal ACE, QoL, self- esteem, and children's QoL, highlighting the need for longitudinal research to clarify temporal changes and causal pathways. Reliance on maternal self-reports may introduce recall and social desirability biases, particularly in retrospective ACE reporting, which can be influenced by current psychological status. The sample's restriction to a single cultural and regional context reduces generalisability to populations with differing values, family structures, and policy environments. Potential confounders such as paternal ACE, marital relationship quality, family conflict, and discrimination experiences were not measured, underscoring the need for future multivariate models encompassing the developmental histories and interactions of all family members. Furthermore, the use of subjective measures for QoL and self-esteem warrants integration of objective health indicators or observational data to enhance interpretative validity. Finally, the control of only a subset of socio- ecological covariates leaves open the possibility of residual confounding from unmeasured factors, including community safety, housing conditions, and access to health and welfare services, which may further influence children's QoL.

## Conclusion

This study, grounded in Bronfenbrenner's ecological systems theory, examined the influence of maternal ACEs on children's QoL and identified the specific pathways involved. Findings indicated that maternal QoL partially mediated the relationship between maternal ACEs and children's QoL, while maternal self-esteem functioned as a protective factor moderating this association. These results suggest that although maternal childhood adversity can negatively impact the developmental and welfare outcomes of the next generation, sufficient maternal psychosocial resources can buffer such effects.

The findings highlight the interplay between individual and environmental factors in shaping intergenerational outcomes, integrating ecological theory to provide a comprehensive framework for understanding both risk and protective mechanisms. Practically, the results reaffirm the importance of addressing parental trauma recovery and enhancing psychosocial support to promote children's healthy development.

Interventions that improve maternal QoL and self-esteem may yield benefits not only for maternal well-being but also for children's QoL. This research delivers a clear message: “Strengthening parents is a pathway to supporting children”.

Future research and practice should adopt multilayered approaches encompassing family, community, and policy-level strategies to establish ACE screening and early intervention systems, develop programs that enhance maternal resilience and parenting efficacy, and expand family-friendly social policies. Such efforts could break cycles of adversity, foster positive developmental legacies, and lay a vital foundation for ensuring the happiness and stability of future generations.

## Data Availability

The datasets presented in this study can be found in online repositories. The names of the repository/repositories and accession number(s) can be found below: https://repository.kihasa.re.kr/.
